# Genetic variation and evolutionary demography of *Fenneropenaeus chinensis* populations, as revealed by the analysis of mitochondrial control region sequences

**DOI:** 10.1590/S1415-47572010005000019

**Published:** 2010-06-01

**Authors:** Xiao Yu Kong, Yu Long Li, Wei Shi, Jie Kong

**Affiliations:** 1 Marine Biodiversity Collection of South China Sea, Laboratory of Marine Bio-Resource Sustainable Utilization, South China Sea Institute of Oceanology, Chinese Academy of Sciences, GuangzhouChina; 2 Liaoning Open Lab of Applied Marine Bioligy, Liaoning Ocean and Fishery Science Research Institute, DalianChina; 3 Mariculture Research Laboratory, Ocean University of China, Education Ministry of China, QingdaoChina; 4 Yellow Sea Institute of Fisheries Research, Chinese Academy of Fisheries Science, QingdaoChina

**Keywords:** *Fenneropenaeus chinensis*, mtDNA, isolation with migration (IM) coalescence, historical demography, population expansion

## Abstract

Genetic variation and evolutionary demography of the shrimp *Fenneropenaeus chinensis* were investigated using sequence data of the complete mitochondrial control region (CR). Fragments of 993 bp of the CR were sequenced for 93 individuals from five localities over most of the species' range in the Yellow Sea and the Bohai Sea. There were 84 variable sites defining 68 haplotypes. Haplotype diversity levels were very high (0.95 ± 0.03-0.99 ± 0.02) in *F. chinensis* populations, whereas those of nucleotide diversity were moderate to low (0.66 ± 0.36%-0.84 ± 0.46%). Analysis of molecular variance and conventional population statistics (*F*_ST_ ) revealed no significant genetic structure throughout the range of *F. chinensis*. Mismatch distribution, estimates of population parameters and neutrality tests revealed that the significant fluctuations and shallow coalescence of mtDNA genealogies observed were coincident with estimated demographic parameters and neutrality tests, in implying important past-population size fluctuations or range expansion. Isolation with Migration (IM) coalescence results suggest that *F. chinensis*, distributed along the coasts of northern China and the Korean Peninsula (about 1000 km apart), diverged recently, the estimated time-split being 12,800 (7,400-18,600) years ago.

## Introduction

Mainly distributed in an area within 118° E to 125° E and 33° N to 40° N, *Fenneropenaeus chinensis*, commonly called the Chinese shrimp or fleshy prawn, is a commercially important species in northern China and Korea (Cha *et al.*, 2002; Liu, 1990, 2003). This species has supported one of the most valuable fisheries in northern China, comprising approximately 80% of total annual shrimp landings, before the collapse of aquacultural enterprise (Liu, 1990). As with many other marine fishery species, the Chinese shrimp has undergone a long-term decline in catches owing to overfishing and habitat destruction. Landings gradually decreased, from 20,000 tons in 1970 to 800 tons in 1997 in the Bohai and Yellow Seas of China.

*F. chinensis* is characterized by long-distance migration and schooling behavior (Deng *et al.*, 1983). Based on the recapture data of artificial stocking, *F. chinensis* from along the northern China seas can be divided into two independent coastal populations. One is the Yellow and Bohai Seas (YB) coast population, more numerous and larger in individual size, and the other from the western Korean peninsula (KW) coast population, with relatively smaller numbers and size (Deng *et al.*, 1990). Every year in late March, mature adults of both populations migrate to spawning grounds along inshore waters and shallow estuaries of the Yellow and Bohai Seas, where they spawn in mid-May. As with many other penaeid shrimps, the postlarvae of *F. chinensis* then drift towards the coastline. During June, they move even further into shallow waters (6 to 10 m deep), where they remain in the so called `nursery grounds' for over two months. By late August to early September, juveniles return to deeper waters, where they mate in October. In mid-November, the shrimps migrate southward to the Yellow Sea overwintering grounds, arriving there in late January. It is worthy to note that overwintering grounds of the two stocks are slightly different,with those from the China Seas being located more eastward, and those from the Korean Peninsula more westward (Deng and Zhao, 1991). These two populations are considered to be reproductively isolated from each other.

During recent years, a major effort has been directed to the understanding of population genetic variation and structure of *F. chinensis*. Several different molecular approaches (allozymes, DNA amplification polymorphism [RAPD], restriction fragment length polymorphism [RFLP], mitochondrial DNA sequencing, microsatellites, etc.) have been applied to the study of genetic variability at the population level (Shi *et al.*, 1999, 2001; Liu *et al.*, 2000a, 2000b, 2006; Wang *et al.*, 2001; Zhuang *et al.*, 2001; Meng *et al.*, 2004; Cui *et al.*, 2007). When compared with other arthropods and penaeids, *viz*., *Litopenaeus setiferus*, *Marsupenaeus japonicus* etc., already extensively studied, the level of genetic diversity of *F. chinensis* has proved to be much lower (Hualkasin *et al.*, 2003; McMillan-Jackson and Bert, 2003, 2004; Tzeng *et al.*, 2004). The results obtained from using the above mentioned markers may be discordant with each other, even taking into account the different populations used for analysis.The datasets of mitochondrial DNA (16S rRNA and cytochrome oxidase I genes) (Quan *et al.*, 2001; Hwang, 1996; Hwang *et al.*, 1997;), PCR-RFLPs of mtDNA control region (Cui *et al.*, 2007) and microsatellites (Liu *et al.*, 2006), revealed either the absence of or negligible genetic differentiation in *F. chinensis* from the Bohai and Yellow Seas. In contrast, *Gst* values calculated from RAPD data revealed significant genetic differentiation between pairs of samples of this species from the same region (*Gst* = 0.032-0.233, Meng *et al.*, 2004).

It has been shown in a *M. japonicus* species complex, that mitochondrial DNA data gave a clearer picture of population differentiation patterns than microsatellites (Tsoi *et al.*, 2007). Yet the mitochondrial 16S rRNA and COI genes used in the previous studies of *F. chinensis* were often empolyed in elucidating population structure of penaeid shrimps over a broad geographic range (Klinbunga *et al.*, 2001; Tsoi *et al.*, 2007), they were too conserved to reveal genetic structure on a small geographic scale (Tsoi *et al.*, 2007), as is the case of *F. chinensis*. Recently, Cui *et al.* (2007) examined *F. chinensis* variation, using mtDNA control region PCR-RFLP data. Actually, the low detectable rate of mutations in PCR-RFLP of the mitochondrial control region (CR) (H = 0.24) was insufficient for detecting significant genetic structure in this species.

Mitochondrial DNA sequencing, particularly of the most rapidly evolving and highly variable control regions, has proved to be a useful tool for population genetic studies of many terrestrial and aquatic organisms (Avise, 1994). So far, population genetic variation and structure of several penaeids species have been examined with mitochondrial control region, including *Fenneropenaeus merguiensis, Farfantepenaeus aztecus, Litopenaeussetiferus, Farfantepenaeus duorarum, Marsupenaeusjaponicus,* (Chu *et al.*, 2003; McMillan-Jackson and Bert, 2003, 2004; Tzeng *et al.*, 2004; Valles-Jimenez *et al.*, 2006; Tsoi *et al.*, 2007). The results indicated that the control region provides more informative sites (with more haplotypes, accordingly), thus making it the most useful region for evaluating genetic variation within and between populations of penaeid shrimp species.

Thus, we analyzed samples of *F. chinensis* from both the Yellow and Bohai Seas using sequence data of the control region to address the following questions: (1) to examine sequence variability and geographic structure of this species, particularly whether the two populations of *F. chinensis* distributed along the coasts of north China and the Korean Peninsula (about 1000 km apart) have diverged; (2) to infer historical population processes (*e.g.*, population fragmentation, range expansion, or long distance colonization), or present-day processes (*e.g.*, restrict gene flow) that might have affected the current distribution of this species.

## Materials and Methods

###  Sample collection

**Figure 1:**
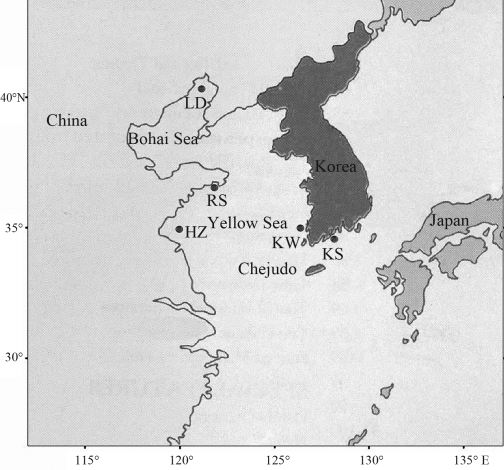
A map showing the sampling locations of *F. chinensis*, which are marked by abbreviations corresponding to those in Table 1.

A total of 93 individuals of *F. chinensis* were collected from five localities over most of its range during 1997 to 2001 (Table 1, Figure1). Fresh muscle tissue from the samples was stored at -70 °C before DNA extraction.

In the east and south China Seas, the species is very rare, whereby samples were only sporadically and rarely available, thereby precluding their inclusion in the study.

###  DNA extraction, amplification and sequencing

Genomic DNA was isolated from muscle tissue using the standard phenol-chloroform method and subsequently re-suspended in 50 μL TE buffer. Mitochondrial control region (CR) sequences were amplified using the primers DLA: 5'-AAGAACCAGCTAGGATAAAACTTT -3' (Chu *et al.*, 2003) and DLB: 5'-GCTTACATGTTCTAC CCTATCAAG -3' (McMillan-Jackson and Bert, 2003). PCR amplification was carried out in an Eppendorf authorized thermal cycler. Reactions were conducted in 25 μL volumes containing 1 U *Taq* DNA polymerase (Takara, China), 0.1 mM primers, 2.0 mM MgCl_2_, 0.1 mM dNTPs and 2.5 μL 10x PCR buffer, with approximately 30 ng of DNA as template. Cycling conditions were as follows: an initial 3 min at 95 °C, followed by 36 cycles of 50 s at 95 °C, 1 min at 50 °C, and 90 s at 72 °C, with a final 5 min at 72 °C. The PCR products were visualized on 1% agarose gels, and purified with a Takara Agarose Gel DNA Purification Kit (Takara, China). The purified products were used as template DNA for cycle sequencing reactions performed using a BigDye Terminator Cycle Sequencing Kit (ver. 2.0, PE Biosystems, Foster City, California), whereas sequencing itself was undertaken on an ABI Prism 3730 (Applied Biosystems) automatic sequencer with both forward and reverse primers. The primers used for sequencing were the same as those for PCR amplification. Control region sequences were deposited in the GenBank database under Accession numbers GQ283010-GQ283077.

###  Data analysis

Sequences from both directions in each specimen were aligned with CLUSTAL X1.81 (Thompson *et al.*, 1997) and individual consensus sequences were retrieved by means of both alignment and manual checks. Molecular diversity indices such as the number of haplotypes, polymorphic sites, transitions, transversions and indels, were obtained using Arlequin (Ver. 2.0, Schneider *et al.*, 2000). Haplotype diversity (H), nucleotide diversity (π), and their corresponding variances were calculated according to Nei (1987), as implemented in Arlequin. Implemented by Modeltest 3.06 (Posada and Crandall, 1998), hierarchical series of likelihood ratio tests (Huelsenbeck and Rannala 1997) were used to identify the appropriate nucleotide substitution models.

The examination of significant population structure in *F. chinensis* was by way of molecular variation analysis (AMOVA, Excoffier *et al.*, 1992). Arlequin was used for AMOVA and bootstrap analysis with 5,000 replicates. For CR data, the appropriate model of nucleotide substitution was HKY85 (Hasegawa *et al.* 1985), with invariable sites and gamma shape parameter (HKY+I+G, I = 0.77,G = 0.61). As the HKY model was not available in Arlequin, the more inclusive Tamura-Nei (TrN) (Tamura and Nei, 1993) model, with the same gamma parameters, was used to calculate genetic pairwise distances between haplotypes. *F*_*ST*_ statistics were estimated for pairs of populations. *F*_*ST*_ significance (5% level) was tested by 1,000 permutations for each pairwise comparison.

Haplotype phylogenetic trees were constructed using PAUP (Swofford, 2002) and Mrbayes 3.1.2 (Ronquist and Huelsenbeck, 2003). A maximum likelihood (ML) strategy was implemented to construct a phylogenetic tree from the maximum likelihood (ML) distances deduced by means of selected models. Branch supports in ML trees were estimated by bootstrap analysis of 100 replicates. Bayesian phylogenetic analysis was initiated with random starting trees and run for 1,000,000 generations. Markov chains were sampled every 100 generations. Of the resulting 10,000 trees, 2500 were discarded as “burn-in.” In addition, genealogical relationships were examined by constructing haplotype networks using the median-network approach (Bandelt *et al.*, 1995, 2000).

The historical demographic expansions were examined by two different approaches. Firstly, the Tajima's *D* test (Tajima, 1989) and the Fu's *Fs* test (Fu, 1997) were used to test whether neutrality holds. Significant negative *D* and *Fs* statistics can be interpreted as signatures of population expansion. Historical demographic expansion was also investigated by examining frequency distributions of pairwise differences between sequences (mismatch distribution), based on three parameters, θ_0_, θ_1_ (before and after population growth) and τ (time since expansion, expressed in units of mutational time) (Rogers and Harpending, 1992; Rogers, 1995). Mismatch distribution is usually multimodal in samples drawn from populations at demographic equilibrium, but is usually unimodal in populations following recent population demographic and range expansion (Slatkin and Hudson, 1991; Rogers and Harpending, 1992; Ray *et al.*, 2003; Excoffier, 2004). Arlequin was used for mismatch analysis and neutrality tests. The Harpending (1994) raggedness test was applied to ascertain whether an observed mismatch distribution is drawn from an expanded population (small raggedness index) or a stationary one (large raggedness index). The parameters of demographic expansion τ, θ_0_ and θ_1_ were estimated by a generalized non-linear least-square approach, and parameter confidence intervals were computed using a parametric bootstrap approach (Schneider and Excoffier, 1999). τ values were transformed to estimated real time since expansion, through the equation τ = 2*ut*, where *u* is the mutation rate per generation for the whole sequence under study and *t* the time measured in years since expansion.

The Hey and Nielsen (2004) IM program was also used to estimate genetic diversity, migration rates and divergence time. The IM-model (isolation with migration) assumes that an ancestral population splits into two populations at a time *t*, and that descendant populations may exchange migrants in both directions at unequal rates (Hey and Nielsen, 2004; Hey, 2005). In practice, through IM implementation it is possible to estimate six parameters during MCMC simulation: the genetic diversity in descendant populations (θ_1,2_ = *N*_e_μ), genetic diversity in the ancestral population (θ_A_ = *N*_e_μ), divergence time (*T* = *t*μ) and migration rates (*m*_12_ = *m/*μ) between the two diverging populations (Hey and Nielsen, 2004). In addition, the program can register an estimate for the most recent common ancestor (TMRCA) for each locus. The IM method can be used for closely related populations or species; here it was applied to closely related populations defined by the tagging study. This should shed light on the geographical distribution of genetic diversity, divergence time of the two populations and their mutual gene flow.

The HKY (Hasegawa *et al.*, 1985) substitution model with inheritance scalar 0.25 was assumed in all IM-runs as recommended for mtDNA data (Hey and Nielsen, 2004). Based on the results from initial runs, parameter boundaries were adjusted according to the location of probability distribution. In each run, the first million steps were discarded before starting recording the parameter values and the runs were continued until the effective sample size was at least 500 for all the parameters. All runs were repeated twice, starting from random parameter values to check whether independent runs converged to similar parameter estimates. Likelihood values for θ, *M*, and *T* were calculated and the values with the highest posterior probability accepted as the best estimates. Values for *N*_ef_, *t*, and TMRCA were calculated using a generation time of 1 year and mutation rates estimated at 1.9 x 10^-7^ substitutions/site/year (McMillan-Jackson and Bert, 2003). Scaling these figures per generation and for 993 nucleotides gave A value of *u* = 1.88 x 10^-4^.

## Results

###  Genetic variation

A 993-bp CR fragment was bidirectionally sequenced for 93 individuals of *F. chinensis*. The average base composition was as follows, A: 37.25%, T: 45.24%, G: 7.93%, C: 9.57%. The high AT content (82.49%) of this sequence was consistent with that of counterpart sequences from other *Penaeus* species (*F. aztecus*: 79%; *L. setiferus*: 83%; *F. notialis*: 79%; *F. duorarum*: 82%). When regions of ambiguous alignment were removed, 84 segregating sites were detected, defining 68 haplotypes, which included 72 transitions, 12 transversions and 3 indels.

The number of detected haplotypes within samples ranged from 15 (KS) to 19 (LD) (Table 2, Table 3). The majority of these (56/68, 82.4%) were sample-specific, *i.e.*, observed in only one location. Two were found in more than one individual, but only in one specific sample locaton, whereas each of the remainder was found in only one individual, respectively*.* Twelve haplotypes were shared among locations, five (11, 12, 16, 23, 28) being coincident on both the coasts of China and the Korean peninsula (Table 2)*.*

Haplotype diversity (*h*), nucleotide diversity (π), and other sample-specific diversity indices are presented in Table 3. Haplotype diversity levels were very high (0.95 ± 0.03-0.99 ± 0.02) for *F. chinensis* populations, whereas those of nucleotide diversity were at a moderate to low level (0.66 ± 0.36%-0.84 ± 0.46%*)*. In addition, on comparing genetic variation with that of several other penaeid species (Hualkasin *et al.*, 2003; McMillan-Jackson and Bert, 2003, 2004; Tzeng *et al.*, 2004; Table 4), it was shown that the variation level of *F. chinensis* is almost at the lowest end of the scale so far.

**Figure 2:**
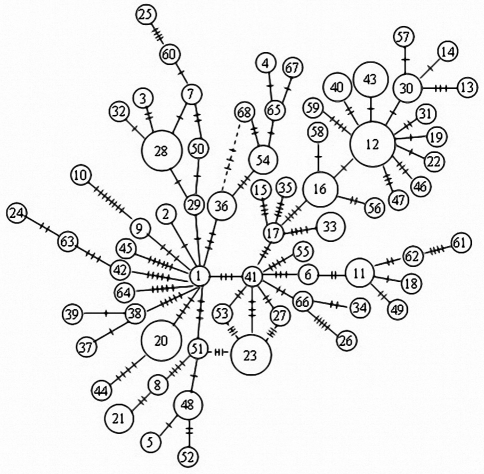
Minimum spanning tree of control region haplotypes in *F. chinensis*. The sizes of the circles are proportional to haplotype frequency. Perpendicular tick marks on the lines linking haplotypes represent the number of nucleotide substitutions.

A minimum spanning tree was constructed based on site differences between all the 68 haplotypes (Figure 2). The twelve haplotypes found in multiple samples occupied a central position in the tree. The remainder were separately derived from those to which they were closely related. Pairwise sequence divergence estimated among the 68 haplotypes varied from 0.001 to 0.0174, with an average of 0.008. Phenograms based on maximum likelihood (ML) distances and Bayesian phylogenetic analysis were consistent with the minimum spanning network, as regards the lack of geographical structure.

###  Population structure

Genetic differentiation among Chinese shrimp populations was assessed through *F*_*ST*_ pairwise comparison. In general, *F*_*ST*_ values were low (-0.02343~0.02112) and none statistically significant (p > 0.05). In the 10 possible comparisons, four of the *F*_*ST*_ estimates were negative (Table 5), this indicating that variation within was greater than between populations. Based on AMOVA analysis, no statistically significant geographical structure was detected among the populations studied (Table 6). When the samples were pooled into two groups: northern China (RS/HZ/LD) and the Korean Peninsula (KW/KS), AMOVA analysis indicated that variation within populations contributed to 99.71% of the total variation. This is even more evident from the haplotype network (Figure 2), in which there is no apparent relationship between the locations where a given sample was located and its genetic relationship with the other haplotype. Therefore, knowing the haplotype of a given individual could be useless in predicting the place of collection.

###  Past population expansion

**Figure 3:**
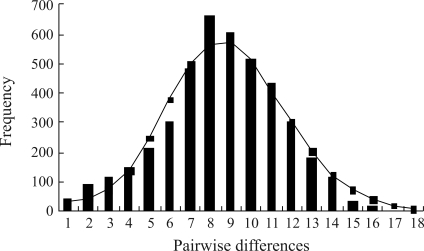
The observed pairwise difference (bars) and the expected mismatch distribution (line) under the sudden expansion model of CR gene haplotypes in *F. chinensis*.

Estimates from Tajima's *D* and Fu's *F*s test were negative in all locations, although associated probabilities did not reach statistical significance in most cases (Table 3). However, on pooling samples, Tajima's *D* and Fu's *F*s results were significant at the 5% level. Furthermore, when analyzing all the samples together, the distribution of pairwise nucleotide differences (mismatch distribution) revealed a smooth unimodal pattern, characteristic of population expansion (Figure 3). The Harpending raggedness index was uniformly low, confirming the satisfactory fit of the data to a unimodal distribution.

Estimates of θ_0_ and θ_1_ indicated that populations expanded from a very small (close to 0 in some cases) to a very large size, with a 95% confidence interval (CI) in all cases (Table 3). The tau value (τ), which reflects the location of the mismatch distribution crest, provided a rough estimate of the time when rapid population expansion started. The observed value of the age expansion parameter (τ) was 8.109 (95% CI: 5.445-9.414). The estimated time of expansion for *F. chinensis* was 21,500 (14,440-25,000) years ago*,* based on a mutation rate of 19%/MY for CR gene (McMillan-Jackson and Bert, 2003) and the equation τ = 2*ut*.

###  IM coalescene

**Figure 4:**
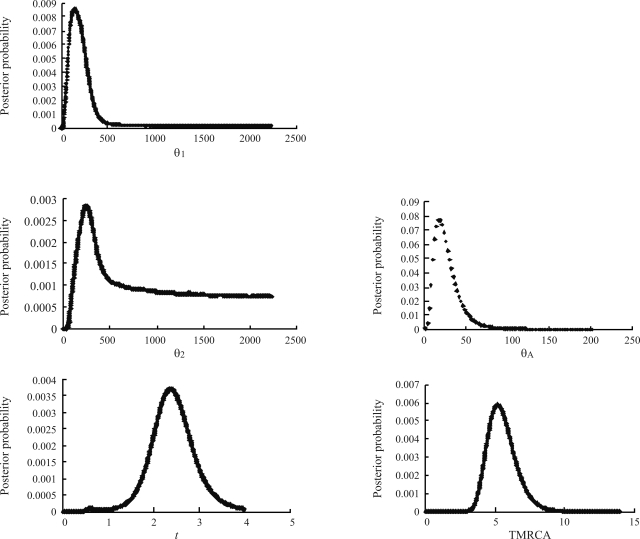
Posterior distribution of parameter estimates from the IM program. θ_1_, θ_2_, and θ_A_ are effective population sizes of the west Korean peninsula (KW) coast population, the Yellow and Bohai Seas (YB) coast population, and the ancestral population; t is the time since population divergence, and TMRCA the time until the most recent common ancestor of the haplotypes.

IM coalescence was used to further examine whether the two populations of *F. chinensis* had diverged and the species expanded. Initially, the samples were pooled into two populations, *i.e.* northern China (RS/HZ/LD) and the Korean Peninsula (KX/KS). Results strongly indicated unimodal posterior distribution at four parameter estimates (θ_1_, θ_A_, *t*, TMRCA). Smoothed θ_1_ posterior distribution peaked at 197.7 (95% HPD: 54.7-1892.8), and that of θ_A_ at 23.5 (95% HPD: 7.8-70.4). However, posterior distributions of θ_2_ were mostly across a broad range of values (Figure 4), the tail of the posterior distribution did not reach zero. Our estimates of population size parameters (θ_1_, θ_2_, θ_A_) suggest that *F. chinensis* has undergone population expansion. The densities of migration rate parameters are fairly flat (Data not shown), thereby indicating that the data contain little information on migration within the framework of an IM model. Since the model assumes a constant rate of gene flow after population separation, it is expected that migration rates between populations that have only recently split, as appears to be the case, would be difficult to estimate. The posterior distribution of *t* (scaled divergence time) peaked at 2.4 (95% HPD: 1.4-3.5), which, when converted to time in years, implies that the two populations began diverging about 12,800 bp (range = 7,400 to 18,600). Our estimate of TMRCA substantially predates these estimated times of divergence, and therefore some of the extant diversity was most likely present prior to population divergence. Posterior probabilities of TMRCA peaked at 5.5, indicating that all the sampled haplotypes coalesced at approximately 29,300 bp.

## Discussion

###  Low levels of population differentiation among *F. chinensis* populations

**Figure 5:**
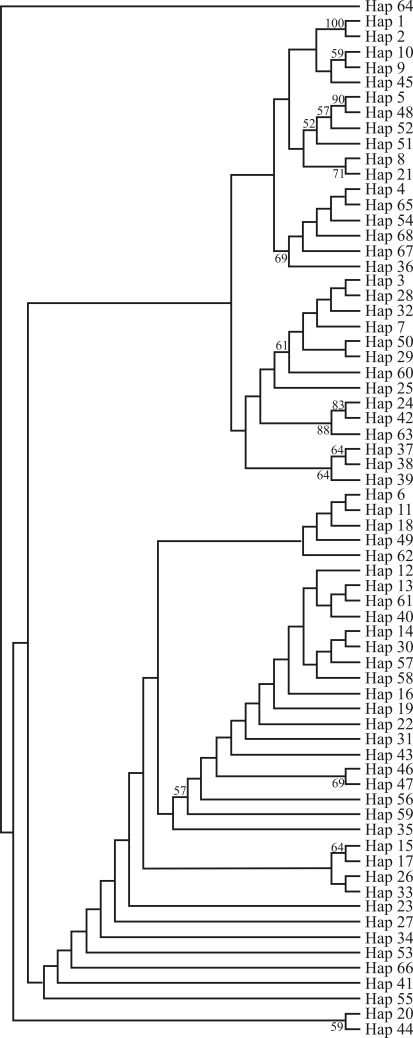
Topology derived from maximum-likelihood (ML) analysis of 68 mitochondrial control region haplotypes, which was built using the HKY85 model with invariable sites and gamma-shaped parameters (HKY+I+G, I = 0.77,G = 0.61). Nodal support indicated by bootstrap values (BP) was presented for ML analysis.

No significant differences at all hierarchical levels were detected through AMOVA analysis. Furthermore, all the conventional *F*_*ST*_ population statistics were insignificant (*F*_*ST*_ values ranged from -0.02343 to 0.02112), thereby indicating that no significant population structure exists throughout the *F. chinensis* range, which is consistent with findings from previous research (Quan *et al.*, 2001, 2007; Liu *et al.*, 2006). This was also obvious in the phylogenetic trees (Figure 5), where populations grouped without any evidence of geographical meaning. Such a lack of population differentiation also occurs in other penaeid species, *i.e.F. aztecus* and *F. duorarum*, but differs from the distinct population structure found in *L. setiferus* (McMillan-Jackson and Bert, 2003, 2004) and *L. vannamei* (Valles-Jimenez *et al.*, 2006).

Two mutually different, but not exclusive, factors might be responsible for this low level of divergence, *i.e.*, contemporary gene flow and/or recent isolation. Theoretically, gene flow at the rate of a few individuals per generation would be sufficient to prevent the accumulation of significant genetic drift between geographically distant locations (Hartl and Clark, 1989). However, stocking and recapture data indicate that the reproductive migration routes and directions of the two populations have become separated from their over-wintering migration, with the result that they are currently geographically and reproductively isolated from each other (Deng *et al.*, 1983, 1990). Moreover, from IM analysis, it can be deduced that *F. chinensis* , as dispersed along the coast of both northern China and the Korean Peninsula (about 1000 km apart), has only recently diverged. The estimated split-time was 12,800 (7,400-18,600) years ago, suggesting that the two populations were recently derived from a single population. Consequently, these mutual and shallow genetic relationships among *F. chinensis* populations are consistent with recent divergences (after the Pleistocene), thereby playing an important role in shaping the current genetic structure of Chinese shrimps.

###  Recent evolution of *F. chinensis* populations

The mitochondrial control region is often used in population studies, due to its high level of polymorphism (Lee *et al.*, 1995). High *h* and moderate π values were clearly observed in the samples of *F. chinensis* (0.95 < *h* < 0.99; 0.0069 π < 0.0084; Table 3). Such a combination of high level of haplotype and moderate to low level of nucleotide diversity in various marine species, has often been attributed to expansion after a period of small effective population size, as rapid population growth enhances the retention of new mutations (Avise *et al.*, 1984; Watterson, 1984). Many of these marine species are believed to have originated in the Pliocene or early Pleistocene, but their mtDNA genealogies coalesced more recently, perhaps during the last few hundred thousand years (Grant and Bowen, 1998). In this study, the estimated TMRCA for Chinese shrimps was 29, 300 years ago based on IM coalescence, thereby indicating mtDNA CR haplotype coalescing at approximately 29,300 bp.

Another indication of recent *F. chinensis* expansion is the Poisson-like distribution of the number of nucleotide differences observed on comparing haplotypes in various populations (Figure 3). This distribution is attributed to mutation-drift disequilibrium caused by explosive population growth (Rogers and Harpending, 1992). Based on theory and computer simulations, Rogers and Harpending (1992) hypothesized that recent genetic bottleneck phenomena result in L-shaped distributions. Subsequent rapid population growth could generate a `wave' in distribution that would propagate to the right over time.

The mismatch distributions observed in the data set studied are clearly unimodal, a result that is consistent with a recent demographic expansion (Slatkin and Hudson, 1991; Rogers and Harpending, 1992). Based on the tau value (τ), the estimated expansion time for Chinese shrimps was 14,440-25,000 years ago. This timescale coincides with events occurring in the late Pleistocene global glacial period (about 10,000-20,000 years ago), with frigid and arid climatic conditions world-wide (Zhu *et al.*, 1998), coinciding with the formation of the Bohai and Yellow Seas during the late Pleistocene marine transgression (Wang, 1980).

From IM-coalescence results, it has also been demonstrated that *F. chinensis* is characterized by recent population expansion. This expansion is bolstered up by the relatively small ancestral population size, in contrast to the large current population size (Figure 4). This increase is likely to have occurred during the late Pleistocene or early Holocene. Significantly negative estimates from Tajima's *D* and Fu's *F*s testing, as well as the star-like shape of the haplotype network, give support to the population expansion hypothesis.

Therefore, our data provided evidence of present-day restricted gene flow in *F. chinensis*. Phylogenetic relationships among regional populations are probably not resolved due to the recentness of divergence. To further tackle this issue, it is desirable to undertake a large-scale tagging study covering the entire geographic range of *F*. *chinensis*, with the combined efforts of a greater number of scientists.

###  Comparison with previous studies

We observed a diversity peak in the samples near the Yellow Sea overwintering-grounds and a decline in others far distant. This pattern was not apparent in previous surveys (with data of allozyme, RAPD, microsatellite, COI, PCR-RFLP), but diploid loci are expected to be less sensitive in this respect, as a four-fold decrease in population size would be needed to show a reduction comparable with that observed in haploid mtDNA. MtDNA diversity gradients most likely emanated from extinction and recolonization in marginal areas. MtDNA diversities in the newly abundant stocks are expected to be reduced, compared with those in neighboring regions.

Most previous studies of the population structure of *F. chinensis* showed lack of genetic differentiation in this species. However, an RAPD analysis (Zhuang *et al.*, 2001) supported the occurrence of two *F. chinensis* geographic populations in the Yellow Sea and the Bohai Sea. In their study, shrimp samples were collected from only three localities, two from the Korean Peninsula and one from the coast of China. The subsequent study by the same group using microsatellites detected little genetic differentiation between the populations (Liu *et al.*, 2006). An appraisal of evolutionary relationships among the three populations indicated the highest degree of genetic identity in the two from the Korean Peninsula, whereas those from the other areas were only distantly related. The author considered it most likely that the wild populations of *F. chinensis*, which is extensively distributed in the Yellow and Bohai Seas, consists of two independent populations. Our data, as presented here, are consistent with this result.

## Conclusions and implications for management

Generally, a single panmictic population is capable of recovering through increased recruitment by propagation (Munro and Bell, 1997). Nevertheless, different populations with a unique genetic structure should each be managed as distinct units, these requiring separate monitoring and management owing to the different levels of gene flow and demographic history (Salgueiro *et al.*, 2003). Therefore, we suggest that *F. chinensis* from the coasts of China and Korea should be treated as separate management units, and fishery management should be conducted separately by regional authorities. Efforts should be made to sustain population stability, since genetic diversity is a prerequisite for evolutionary adaptation to a changing environment (Hedrick and Miller, 1992).

Our work also shows the potential of coalescence-based models in inferring evolutionary processes, and illustrates the value of a historical perspective in developing a full understanding of contemporary genetic patterns and in designing adequate conservation strategies. On considering that historical population expansion masks low migration, with the subsequent tendency to upgrade gene flow estimates, the low level of differentiation between localities as detected could be explained accordingly.

## Figures and Tables

**Table 1:** The information on *F. chinensis* samples used in this study.

Locality	Abbrev.	Collection date	Sample size
West coast of the Korean Peninsular (126° E, 35° N)	KW	May, 1997	17
Nan Hai along the shore of Korea (34°30' N, 127°30' E)	KS	Sept. 2005	21
Rushan Bay of the Yellow Sea (122° E, 37° N)	RS	Aug. 2001	17
Liaodong Bay of the Bohai Sea (40°30' N, 121°30' E)	LD	Sept. 2001	20
Haizhou Bay of Yellow Sea (35° N, 120° E)	HZ	Sept. 2001	18

**Table 2:** The distribution of all haplotypes in *F. chinensis* among the five locations.

Location	Haplotype																																	
	01	02	03	04	05	06	07	08	09	10	11	12	13	14	15	16	17	18	19	20	21	22	23	24	25	26	27	28	29	30	31	32	33	34
KW	1	1	1	1	1	1	1	1	1	1	1	2	1	1	1	1																		
KS												1				1	1	1	1	4	2	1	3	1	1	1	1	1	1					
RS																												1		1	1	1	1	1
HZ																							1					2					1	
LD											1	2				1														1				

	35	36	37	38	39	40	41	42	43	44	45	46	47	48	49	50	51	52	53	54	55	56	57	58	59	60	61	62	63	64	65	66	67	68

KW																																		
KS																																		
RS	1	1	1	1	1	1	1	1	2	1																								
HZ						1					1	1	1	1	1	1	1	1	1	1	1	1	1											
LD		1							1					1						1				1	1	1	1	1	1	1	1	1	1	1

For sample abbreviations see Table 1.

**Table 3:** Summary of molecular diversity in *F. chinensis*. Corresponding Tajima's *D* and Fu's *F*s P values and mismatch distribution parameter estimates are also indicated.

Population	No. of haplotype				Tajima's *D*			Fu's *F*s			Mismatch distribution		
		*S*	*h*	π (%)	*D*	p		*F*s	p		τ	θ_0_	θ_1_
KW	16	39	0.99 ± 0.02	0.8258 ± 0.4508	-1.06	0.14		-7.74	0.003		9.172	0.03	62.63
KS	15	31	0.95 ± 0.03	0.6938 ± 0.3795	-0.78	0.23		-4.16	0.039		7.521	0	99999
RS	16	32	0.99 ± 0.02	0.7742 ± 0.4249	-0.77	0.25		-8.17	0.001		8.346	0	99999
HZ	17	39	0.99 ± 0.02	0.8375 ± 0.4551	-1.04	0.17		-8.85	0.000		7.277	0	99999
LD	19	32	0.99 ± 0.02	0.6626 ± 0.3649	-0.97	0.17		-12.65	0.000		7.791	0	44.76
Total	68	84	0.99 ± 0.004	0.7598 ± 0.3964	-1.79	0.01		-24.85	0.000		8.109	0	114.34

*S*, number of segregating site; *h*, haplotype diversity; π, nucleotide diversity.

**Table 4:** Comparision of genetic parameters in six *Penaeus* species.

Species	mtDNA marker	Sequence divergence %	Nucleotide diversity (π) %
*F. aztecus*	CR	0.20-5.80	2.10
*L. setiferus*	CR	0.20-3.20	1
*F. duorarum*	CR	0-6	-
*M. japonicus*	CR	-	2.51 ± 0.70
*F. notialis*	CR	0.2-3.9	1.8
*F. chinensis*	CR	0.1-1.74	0.7598 ± 0.3964

**Table 5:** Genetic differentiation in five populations of *F. chinensis*.

Population	KW	KS	RS	HZ	LD
KW		n.s	n.s	n.s	n.s
KS	0.00328		n.s	n.s	n.s
RS	0.00957	0.01309		n.s	n.s
HZ	-0.02343	0.00336	-0.00542		n.s
LD	0.00007	0.02112	-0.00947	-0.01122	

n.s, not significant; *F*_ST_values are below the diagonal and P values are above the diagonal.

**Table 6:** Analysis of molecular variation in populations of *F. chinensis*.

	Degree of freedom	Summation of mean square	Contribution of variation	Percentage of variation	*F*_ST_	p
Among populations	4	14.976	-0.00024	-0.01	-0.00006	0.44
Within populations	88	329.854	3.74834	100.01		
Total	92	344.83	3.74810	100.00		
